# La prise en charge des suicides par précipitation en traumatologie

**Published:** 2010-09-17

**Authors:** Ibrahimi Abdelhalim El, Shimi Mohammed, Daoudi Abdelkrim, Elmrini Abdelmajid

**Affiliations:** 1 Service de chirurgie ostéo-articulaire B4, CHU HASSAN II de Fès, Maroc

**Keywords:** Suicide, tentative de suicide, Maroc, Psychiatry, trauma

## Abstract

**Introduction:**

A travers ce travail, l’épidémiologie et la psychopathologie du suicide par précipitation seront rappelées, le rôle du chirurgien traumatologue dans la restauration physique et psychique sera démontré.

**Méthodes:**

Nous rapportons une étude rétrospective de 15 suicidants par précipitation admis pour des traumatismes complexes de l’appareil locomoteur entre 2004 et 2007.

**Résultats:**

Il s’agissait de primosuicidants, comprenant 10 femmes et 5 hommes. L’âge moyen des hommes est de 26,5 ans (24-34 ans) et celui des femmes de 40,7 ans (17-45 ans). Le bilan lésionnel des suicidants a retrouvé 14 fractures des membres inférieurs, 4 traumatisme du rachis avec compression médullaire dans 1 cas, une fracture du bassin; deux traumatismes du membre supérieur. Aucun de ces patients n’a présenté de lésions viscérales ni de traumatismes crâniofacial. 80% ( n = 12) des patients souffraient d’une affection psychiatrique avant leur passage à l’acte suicidaire : 8 pour une pathologie dépressive et 4 pour une schizophrénie. Chaque suicidant a subi au moins un acte chirurgical. Tous les patients avaient poursuivi la prise en charge psychiatrique en ambulatoire. Nos résultats fonctionnels étaient satisfaisants sans complications. Aucun des patients n’a récidivé. La pathologie suicidaire représente un problème de santé publique. Le suicide par précipitation représente 5 % de l’ensemble des modalités suicidaires. Les traumatismes occasionnés par ce mode suicidaire sont complexes et graves. Leur prise en charge nécessite une collaboration multidisciplinaire.

**Conclusion:**

La réparation chirurgicale permet à la fois le rétablissement de la fonction et l’amélioration psychique du sujet prévenant contre une rechute dépressive ou une récidive suicidaire.

## Introduction

La pathologie suicidaire représente un problème de santé publique. Le suicide par saut ou par précipitation apparaȋt comme un mode particulier de suicide tant par sa faible fréquence puisque il ne représente que 5 % des suicides ainsi que par sa gravité [1].

Sa rareté et son caractère spectaculaire qui a un fort impact sur l’opinion publique, expliqueront le peu d’études concernant les modalités suicidaires ainsi que celles consacrées aux survivants lorsque la précipitation a été le premier mode suicidaire utilisé.

Les traumatismes occasionnés par le suicide par saut sont complexes et graves, leur prise en charge nécessite une collaboration multidisciplinaire.

Dans ce travail, nous avons suivi des patients ayant survécus à leur geste suicidaire par précipitation et présentant des traumatismes complexes, pour tenter de mieux cerner leur psychopathologie, préciser le rôle du chirurgien, qui permettrait à la fois le rétablissement de la fonction et l’amélioration psychique du sujet, prévenant contre une rechute dépressive ou une récidive suicidaire.

## Méthodes

Nous avons admis 15 sujets primosuicidants, comprenant 10 femmes et 5 hommes. L’âge moyen des hommes est de 26,5 ans (24-34 ans) et celui des femmes de 40,7 ans (17-45 ans). En ce qui concerne la situation martiale, 12 patients sont célibataires, 3 sont mariés. Les hommes sont tous célibataires ( n = 5), les femmes le plus souvent (n= 7). Au niveau professionnel, au moment du geste la majorité sont inactifs n= 11, trois actifs, et 1 étudiant.

**Les caractéristiques du saut**

La hauteur de la précipitation la plus élevée de notre étude est de 22 m; lorsque les patients se sont jetés d’une fenêtre d’immeuble ou d’un balcon, 7 l’ont fait à partir du 1er étage, 5 à partir du 2e étage et un d’un minaret de mosquée.

Le bilan lésionnel des suicidants a retrouvé 14 fractures des membres inférieurs, 4 traumatismes du rachis avec compression médullaire dans 1 cas, une fracture du bassin et deux traumatismes du membre supérieur. Il n’a pas été noté de spécificité de lésions en fonction du sexe. Les lésions osseuses sont donc prédominantes en particulier au niveau des membres inférieurs et du rachis, ce qui indique que la réception au sol s’est fait sur les pieds et vient conforter l’intention suicidaire de la chute. À noter qu’aucun de ces patients n’a présenté de lésions viscérales ni de traumatisme crânio-facial.

L’étude des antécédents psychiatriques montre que 80% des sujets (n=12) souffraient d’une affection psychiatrique avant leur passage à l’acte suicidaire: 8 pour une pathologie dépressive et 4 pour une schizophrénie. Cinq patients sur 15 avaient été hospitalisés antérieurement en milieu psychiatrique et 4 sur 15 recevaient des soins pour une pathologie psychiatrique en ambulatoire.

Nous avons recherché d’autres facteurs qui auraient pu favoriser le passage à l’acte par précipitation. On a trouvé la notion d’alcoolisme chronique chez deux patients qui étaient en état d’ébriété au moment du passage à l’acte suicidaire. Seule une patiente âgée de 24 ans, dépressive chronique avait pris des médicaments avant de se précipiter. Les drogues psychodysleptiques (cannabis…) sont connues pour provoquer un trouble du jugement et une distorsion de la perception; des précipitations ont été décrites soit dans le cadre de fantasmes de vol et d’émancipation de l’apesanteur, soit dans le cadre d’un raptus suicidaire lors d’un mauvais voyage. Un seul patient dans notre étude semble avoir recours à ce type de drogues.

Au total, 20% des patients (3 sur 15) ont associé une prise de toxiques et d’alcool à la précipitation. La répartition des principaux diagnostics psychiatriques posés après le geste suicidaire est illustrée sur le Tableau 1.

**Après la tentative de suicide par précipitation**

Les suicidants de cette présente étude ont subi au moins un acte chirurgical. Tous les patients ont bénéficié d’une consultation psychiatrique en urgence. Concernant l’évolution psychiatrique, aucun des patients n’a récidivé. La prise en charge psychiatrique a été poursuivie en ambulatoire après le séjour dans le service de chirurgie orthopédique. Nos résultats fonctionnels étaient satisfaisants sans complications.

## Discussion

**Epidémiologie**

Le suicide par saut ou par précipitation dans le vide est un mode rare dans la mesure où il ne concerne que 5 à 7 % de l’ensemble des suicides. La rareté du phénomène, dont le caractère « spectaculaire a cependant un fort impact émotionnel sur l’opinion publique, expliquerait le peu d’enquêtes spécifiques disponibles [1].

En revanche, ce mode opératoire est plus répandu dans certains pays que dans d’autres. Au Japon, la projection dans le vide est la méthode la plus utilisée pour se suicide après la pendaison, alors qu’aux états-Unis, les méthodes les plus répandues sont l’utilisation d’une arme à feu, la pendaison et l’overdose 2]. De même, le taux de suicide par saut a tendance à s’accroȋtre dans certains pays. En Malaisie, l’incidence du suicide par précipitation d’un lieu élevé a doublé au cours de ces deux dernières décennies [3]. En France, un rapport de la Direction de la Recherche des études de l’évaluation et des Statistiques [4] indique que le suicide par précipitation fait partie des modes opératoires les moins étudiés dans la mesure où il représente moins de 7 % des suicides. Dans notre étude une prédominance de sujets jeunes a été notée, ce qui a été rapporté par plusieurs auteurs [5,6]. L’étude rétrospective réalisée par Pommereau [7] sur 110 sujets précipitants montre que la proportion de célibataires (45 %) et d’inactifs (61 %) est largement supérieure chez les sujets précipitants en comparaison avec un groupe de suicidants ayant eu recours à une intoxication médicamenteuse volontaire (31 et 33 %). Au niveau sociodémographique, il semblerait donc que le suicide par précipitation témoigne d’une certaine spécificité dans la mesure où il concernerait plus fréquemment des hommes d’âge mûr, célibataires, peu qualifiés sur le plan professionnel et sans emploi.

Dans notre série, 80% des patients présentent des antécédents psychiatriques au sens large. Ce fort pourcentage se rapproche de celui constaté dans les suicides par précipitation lors des autopsies psychologiques décrites par Bourgeois [5].

Les données de la littérature regroupant l’ensemble des cas de précipitation mentionnent en général une prédominance de patients présentant des psychopathologies sévères (schizophrénie, maladie dépressive majeure) [8-11]. Cependant, certains auteurs n’ont trouvé aucune différence entre les groupes de suicidants quant à leur psychopathologie [12]. Les résultats de notre étude sont différents puisque nous avons noté une fréquence plus élevée des troubles de l’humeur dans les antécédents des patients.

**Psychopathologie**

Pour expliquer le choix du mode suicidaire plusieurs hypothèses peuvent être avancées. La précipitation est un mode accessible puisqu’il ne nécessite aucun préparatif particulier ; elle est donc «à la portée de tout le monde», ce qui n’est pas forcément le cas pour d’autres méthodes (acquisition d’une arme à feu, de médicaments…).

*Sur le plan socioculturel*, ce sont les Asiatiques qui se suicident plus souvent par précipitation (et par arme blanche), les Maghrébins utilisent plutôt les caustiques, les Françs la pendaison, les Américains les armes à feu. *Sur le plan symbolique*, Elle peut être liée au monument ou au site, qui peuvent constituer un « attrait » représentatif pour certains suicidants. Ces facteurs interviennent probablement dans un choix qui répond d’abord à la problématique du suicidant : s’infliger une souffrance physique, chercher au contraire à l’éviter, préserver ou au contraire attaquer violemment l’intégrité physique sont autant de soubassements conscients ou inconscients qui jouent un rôle fondamental [13].

**Prise en charge chirurgicale**

La restauration fonctionnelle pourrait être considérée comme un acte de renaissance, de restauration narcissique et de remaniement identitaire. En effet, nous pensons que la prise en charge chirurgicale, impliquant l’acte et la relation avec le chirurgien, participe à l’amélioration psychique du suicidant, en permettant sa reconstruction mentale.

Le rôle du chirurgien ne se réduit pas seulement à l’aspect technique qui est de «rendre la fonction locomotrice», mais il est d’aider le sujet à «faire face» à sa nouvelle vie. Il peut être assimilé à une fonction maternante. Le chirurgien devient en quelque sorte le personnage central, Il est considéré par le patient comme celui qui est capable de lui redonner sa fonction et une nouvelle apparence. Comme le fait la mère vis-à-vis de son enfant, l’image du corps reflétée dans le regard du chirurgien est probablement structurante pour l’identité du patient.

Le statut de suicidant implique une surveillance plus attentive, et son impotence fonctionnelle et ses blessures lui fait bénéficier de multiples soins, allant du nursing aux multiples ostéosynthèses, à des greffes osseuses, cutanées ou à des techniques de reconstruction novatrices. Une telle intensité d’investissement médical et paramédical et d’assistance apporte une revalorisation narcissique au sujet et rehausse à ses yeux la portée sociale du geste suicidaire. Cette prise en charge institutionnelle impose au patient une régression avec l’alitement et les soins, rappelant les soins maternels.

La relation de dépendance du sujet avec l’équipe soignante est au départ presque fusionnel. L’amélioration physique et l’évolution favorable des blessures entraȋnent une diminution progressive des soins, et donc une dépendance moins importante. Parallèlement, la fonction se rétablit, les membres vont subir quelques modifications anatomiques, avec des changements de peau ou l’ajout d’éléments d’ostéosynthèses, de greffes provenant d’autres parties du corps.

Le regard du chirurgien renvoie au sujet un nouveau corps dont il est en règle satisfait. Il existe alors une gratification réciproque. Le patient renaȋt aux yeux du chirurgien, puis aux yeux des autres. Pour Atallah [14], l’anesthésie générale subie par le sujet est considérée comme un état de mort transitoire, cette expérience de mort temporaire est suivie d’une phase postcritique une nouvelle vie et une nouvelle dynamique subjective et relationnelle. Ainsi, par l’impact narcissique qu’offrent les réparations chirurgicales, des remaniements psychiques positifs et notamment identitaires sont facilités.

## Conclusion

Le suicide par saut apparaȋt comme une modalité suicidaire survenant dans des conditions environnementales spécifiques Nous pensons que la réparation chirurgicale permet un travail de reconstruction physique et psychique. Cette étude souligne la nécessité d’une collaboration entre l’équipe chirurgicale du service de traumatologie et le psychiatre dans la prise en charge des suicidants: le psychiatre va permettre par la parole, initialement difficile, une inscription de l’acte suicidaire dans l’histoire du patient, autre que la trace corporelle. Il apporte un soutien non seulement au sujet, mais aussi à la famille et aux soignants; le rôle du chirurgien apparaȋt primordial car il est assimilé à une fonction de renaissance. L’ensemble de cette prise en charge permet la restitution de la dimension d’être, ce qui peut expliquer la rareté des récidives suicidaires.

## Conflits d’intérêts

Les auteurs ne déclarent aucun conflit d’intérêts.

## Contribution des auteurs

**AEI, AD, AE** ont opéré les patients; **MS** a contribué à la recherche bibliographique. Tous les auteurs ont lu et approuvé la version finale du manuscrit.

## Figures and Tables

**Tableau 1:** répartition des diagnostics psychiatriques après le geste suicidaire

**Diagnostics**	**Hommes**	**Femmes**	**Total**
Schizophrénie	4	0	4
Troubles de l’humeur	2	5	6
Troubles de la personnalité	0	3	3
Alcoolisme chronique	2	0	2
